# Editorial: Emotional lateralization and psychopathology

**DOI:** 10.3389/fpsyt.2023.1231283

**Published:** 2023-06-28

**Authors:** Guido Gainotti, Julian Paul Keenan

**Affiliations:** ^1^Institute of Neurology, Catholic University of the Sacred Heart, Fondazione Policlinico Gemelli, Rome, Italy; ^2^Laboratory of Cognitive Neuroimaging, Montclair State University, Montclair, NJ, United States

**Keywords:** aterality, emotion, brain, psychopathology, self-conscious

Different models have been advanced to explain hemispheric asymmetries for emotions (1), and some authors have proposed a strong relationship between emotional laterality and psychopathology. For instance, the “emotional valence” model has led Robinson et al. (2) to suggest that Post-stroke Depression (PSD) may prevail in patients with left-sided lesions, owing to disruption of positive emotions subtended by the left hemisphere. This assumption has been disproven by more controlled investigations (3) and by the systematic review of the predictors of PSD published in this Research Topic by Ladwig et al. who have shown that PSD is mainly due to an interaction between predisposing genetic/epigenetic factors (previous mental disorders) and stroke-related psycho-social factors (physical disability and poor social support). A more general relationship between emotional lateralization and psychopathology has been proposed by an important research group (4), which has suggested that stress may affect hemispheric asymmetries. This hypothesis, however, has not been strongly supported by a study published by Berretz et al. in this Research Topic. In another contribution, Berretz et al. remind us of the evolutionary importance of emotional lateralization because dual processing at an emotional level allows for rapid responses with minimal cognitive appraisal and because both stress and disgust are “experienced” as general adaptive mechanisms.

Another interesting alternative model, also suggesting a strong relationship between emotional lateralization and psychopathology, has been proposed in this Research Topic by Schiffer, who claims that a dramatic personality change is observed after unilateral brain stimulation. This model, however, assumes that emotional lateralization can be observed at the individual level rather than at the population level. This is in contrast to a large body of data showing that a right lateralization of emotion can be observed at the population level and concerns (a) both negative and positive emotion; (b) different aspects of emotion, such as the processing of emotional stimuli at the level of the amygdala, the experience of emotion in the anterior insula, and the control of the emotional response in the ventromedial prefrontal cortex (5). Furthermore, Gainotti (6) has recently shown that the main features of emotional processing (such as its automatic and unconscious nature) are also shared by other activities that are mainly subtended by right hemisphere structures.

We, therefore, believe that only some aspects of psychopathology are influenced by the right lateralization of emotion and that these aspects concern activities at the intersection between emotion and other functions that are mainly subtended by the right hemisphere. Since emotional evaluation is not objective but self-referential (7), psychopathological disorders based on the construct of “self” have often been found in patients with right hemisphere lesions. Clearly related to right hemisphere damage (8) are, indeed, the “delusional misidentifications” in which the patient believes that a familiar person is an impostor (Capgras syndrome) or that a stranger is a known person (Fregoli syndrome). The role of the right hemisphere in these syndromes is probably due to its dominance in the generation of “familiarity feelings” in the presence of known persons (9) and in the recognition of persons by face and voice (10). Consistent with this line of thinking are the results of Keenan et al. (11) and Feinberg and Keenan (8). The former found right frontal activation when contrasting, in an fMRI study, photographs of the self-face with the face of a famous person, whereas the latter commented on these results by suggesting that the right frontal region may play a crucial role in establishing the appropriate relationship between the self and the world. Furthermore, in disorders that involve negative self-assessment (e.g., eating disorders), cortical networks lateralized to the right hemisphere are often implicated (12). Narcissism embodies socio-emotional aspects of self- and other-evaluation, including both an overly positive and negative self-appraisal, in addition to a lack of empathy for others. The right anterior insula appears to be a critical contributor to the disorder (13). A follow-up brain stimulation study revealed that the right dorsal lateral prefrontal cortex, either independently or through connections to the insula, is also a moderator of narcissistic tendencies (14). Lack of empathy is well mapped out in the right hemisphere, which accounts for a large affective component of narcissism (15).

Emotions play a large role in almost all psychopathological conditions. Therefore, having disturbances labeled “Affective Disorders” should not negate the notion that affect is usually involved in any psychological disturbance, whether excessive or restricted. Because emotional expression and recognition are themselves typically lateralized, it is not surprising to see asymmetry in psychopathology.

## Author contributions

GG wrote the first draft of the manuscript and contributed to the conception and design of the Research Topic. All authors contributed to revisions of the manuscript and read and approved the submitted version.
